# A medium‐scale assay for enhancer validation in amniotes

**DOI:** 10.1002/dvdy.24306

**Published:** 2015-08-12

**Authors:** Jingchen Chen, Andrea Streit

**Affiliations:** ^1^ Department of Craniofacial Development and Stem Cell BiologyDental Institute, King's College LondonLondonUnited Kingdom

**Keywords:** conserved regulatory elements, electroporation, green fluorescent protein (GFP), otic placode, PCR, regulatory regions, reporter plasmid

## Abstract

**Background:**

Enhancers are key elements to control gene expression in time and space and thus orchestrate gene function during development, homeostasis, and disease. Whole genome approaches and bioinformatic predictions have generated a tremendous pool of potential enhancers, however their spatiotemporal activity often remains to be validated in vivo. Despite recent progress in developing high throughput strategies for enhancer evaluation, these remain mainly restricted to invertebrates and in vitro cell culture.

**Results:**

Here we design a medium‐scale method to validate potential enhancers in an amniote embryo, the chick. Using a unique barcode for different reporter vectors allows us to detect the activity of nine separate enhancers in a single embryo by one‐step RT‐PCR. The assay is sufficiently sensitive to expand its capacity further by generating additional barcoded vectors.

**Conclusions:**

As a rapid, sensitive, and cost‐effective way to assess enhancer activity in an amniote vertebrate, this method provides a major advance and a useful alternative to the generation of transgenic animals. *Developmental Dynamics 244:1291–1299, 2015*. © 2015 The Authors. Developmental Dynamics published by Wiley Periodicals, Inc. on behalf of American Association of Anatomists

## Key Findings

- Design of a new strategy for rapid enhancer validation in an amniote embryo, the chick.
- Generation of a simple vector for rapid cloning.
- The activity of many enhancers can be detected in a single embryo using a PCR‐based strategy.
- The assay is sufficiently sensitive to detect activity in a small fraction of cells.

## Introduction

Enhancers are *cis*‐regulatory DNA sequences, which increase the expression of their target genes. They are usually located distal to the transcription start site (TSS), but are also found within introns or downstream; they function irrespective of their orientation, distance, and location with respect to the TSS. Enhancers harbour a high density of transcription factor binding sites, and their interacting factors are thought to enhance transcription by interacting with the general transcriptional machinery in the promoter region. The transcription of most genes is regulated by multiple enhancer elements. Their dynamic activity ensures accurate spatial‐temporal expression of their targets during development, homeostasis, and disease and other biological processes. Recent evidence suggests that enhancer regions are flanked by “active marks” on histone tails like H3K27ac (Creyghton et al., 2010; Ernst and Kellis, 2010) and H3K4me1 (Cui et al., 2009; Heintzman et al., 2009; Ernst and Kellis, 2010), as well as being associated with the histone acetyltransferase P300 (Heintzman et al., 2007; Visel et al., 2009). In addition, enhancers are depleted of nucleosomes to provide access for interacting transcription factors (He et al., 2010; Andreu‐Vieyra et al., 2011). Based on these features, chromatin immunoprecipitation using antibodies against H3K27ac, H3K4me1, and/or P300 followed by sequencing, DNase hypersensitivity assays, Formaldehyde‐Assisted Isolation of Regulatory Elements (FAIRE)‐seq, or their combination have identified tens of thousands of potential enhancers in developing organs, e.g., the heart (May et al., 2012), in embryonic stem cell–derived neural crest cells (May et al., 2012; Rada‐Iglesias et al., 2012), and in different human and murine cell human types. The Encode project has contributed a large collection of such enhancers (Consortium, 2012).

Despite the large number of genome‐wide results, not all enhancers have been identified and the tissue‐specificity of many enhancers remains unknown, while others turn out to be false positive (Bonn et al., 2012). To validate a large number of candidate enhancers, low‐cost and time‐effective methods are necessary. Traditionally, enhancer activity is assayed using transgenic approaches in *Drosophila* (McCall et al., 1994), zebrafish (Parinov et al., 2004; Bessa et al., 2009), or mouse (Pennacchio et al., 2006) where reporter constructs are introduced, in which candidate enhancers are cloned upstream of a minimal promoter followed by a reporter like fluorescent proteins or β‐galactosidase. For example, the activity of 1,154 enhancers was validated in different organs during embryonic development and deposited in the VISTA Enhancer Browser (http://enhancer.lbl.gov/). Although this approach provides high spatiotemporal resolution of enhancer activity using, e.g., fluorescent imaging, generation of transgenic animals is labour intensive and costly, and thus not ideally suited for large‐scale enhancer validation. Luciferase reporter assays in cell culture (Nordeen, 1988) are more efficient, however may not recapitulate in vivo enhancer activity.

Recently, several methods for large‐scale enhancer validation emerged, but none was applied to vertebrates due to the bottleneck of generating transgenic animals. Massive parallel reporter assays have recently been developed to allow simultaneous analysis of thousands of reporter plasmids (Patwardhan et al., 2012; Kheradpour et al., 2013). In this assay, the candidate enhancer is placed upstream of a minimal promoter with a unique DNA barcode downstream and a pool of reporter plasmids is introduced into cells. Barcode‐containing transcripts are then sequenced and quantified by deep sequencing. This method provides a powerful tool to dissect the functional nucleotides or motifs within identified enhancers. However, the length of DNA fragments that can be analysed is limited, because the method relies on chemical synthesis to generate them. A similar strategy is employed in sea urchin, where many eggs can easily be injected with a pool of reporter constructs; embryos are screened for fluorescence and the expressed barcodes are quantified by NanoString and RT‐qPCR (Nam and Davidson, 2012). Finally, a large‐scale in vivo method was recently reported in *Drosophila melanogaster* (Gisselbrecht et al., 2013). Transgenic flies are generated with a pool of enhancer‐EGFP constructs. Transgenic animals containing GFP‐expressing cells are then crossed with lines harbouring cell‐type‐specific markers. Double positive cells are selected by FACS and the genomic DNA isolated for deep sequencing to identify cell‐type‐specific enhancers. Despite these successes, it is difficult to apply these methods to vertebrate embryos due to the difficulty to generate transgenic animals with a pool of a large number of different DNA molecules.

Here we developed a customised method for rapid enhancer validation using the chick as an amniote model system. The chick embryo is easily accessible, cost efficient, and lends itself to widespread electroporation for gene transfer and rapid analysis of reporter activity within a few hours after electroporation (Uchikawa et al., 2003; Barembaum and Bronner‐Fraser, 2010; Sato et al., 2010; Betancur et al., 2011). Chick embryos can be grown in ovo as well as outside the egg, and are therefore adaptable for different experimental paradigms. Using a barcoding strategy, we developed vectors to test up to 9 putative enhancers in a few hundred cells in a single embryo. The method can easily be expanded to provide additional barcoded vectors if required. The entire procedure is time‐ and cost‐effective, involving electroporation of plasmid DNA into the chick followed by one‐step RT‐PCR. Results can easily be obtained within two days without expensive reagents and equipment. Assuming that 5–10 different assays can be performed in parallel, this strategy makes the validation of hundreds of putative enhancers possible in amniotes in a relatively short time at low cost.

## Results

### Modification of the pTK‐EGFP Vector

**Figure 1:**
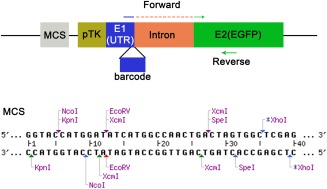
Diagram showing the modified pTK‐EGFP vector. DNA barcodes (16 bp) are inserted at the 3′ end of the first exon (E1), separated by an intron from the EGFP coding sequence in exon 2 (E2). The forward primers for RT‐PCR span the first and second exon consisting of the 16‐bp barcodes and 4 bp of the 5′ end of E2. The common reverse primer is located within the EGFP coding sequence. The modified multiple cloning site contains two extra unique restriction sites, *EcoRV* and *SpeI*, and two *XcmI* sites, which produce a 3′ T overhang after *XcmI* digestion.

In chick, gene transfer is easily achieved using several square pulses of low voltage to electroporate plasmid DNA into target cells. Depending on size and shape of the electrodes, electroporation leads to widespread expression of the transgene. Traditionally, detection of several enhancer constructs in a single embryo is limited by the availability of different fluorescent reporter proteins and relatively high concentrations of plasmid DNA are required for detection by fluorescent microscopy. To increase the capacity, sensitivity, and efficiency of enhancer validation, we modified one of the standard reporter vectors, the pTK‐EGFP vector, which is widely used in chick (Uchikawa et al., 2003). The original vector contains a minimal TK promoter, the first exon transcribing a 5′UTR, followed by an intron and a second exon, which encodes EGFP (Fig. 1). Potential enhancers are inserted into the multiple cloning site (MCS) upstream of the minimal promoter. We introduced two important modifications in this vector. First, we introduced a barcode to generate 9 different vectors: we replaced 16 nucleotides (nt) upstream of the first exon‐intron junction with 9 different 16‐bp barcodes, thus generating 9 vectors each containing a unique barcode (Fig. 1 and Table 1).

**Table 1:** Primers Used to Generate Barcoded Vectors^a^

	Primers	Comments
>T1F1	5′‐ CAGTTTTCAAGCCGGAgtaagtatcaaggttacaagacag ‐3′	For vector with barcode 1
>T1R1	5′‐ TCCGGCTTGAAAACTGacgaccaacttctgcagttaag ‐3′	
>T2F1	5′ ‐ TGATACACCGAGTCGTgtaagtatcaaggttacaagacag ‐3′	For vector with barcode 2
>T2R1	5′ ‐ ACGACTCGGTGTATCAacgaccaacttctgcagttaag ‐3′	
>T3F1	5′‐ AGCTCTTCGCAAAGTGgtaagtatcaaggttacaagacag ‐3′	For vector with barcode 3
>T3R1	5′‐ CACTTTGCGAAGAGCTacgaccaacttctgcagttaag	
>T4F1	5′‐ CAGCTTACTCGTAAGGgtaagtaagtatcaaggttacaagacag ‐3′	For vector with barcode 4
>T4R1	5′‐ CCTTACGAGTAAGCTGacgaccaacttctgcagttaag ‐ 3′	
>T5F1	5′‐ ACGATGAAGCCTTGTCgtaagtaagtatcaaggttacaagacag ‐3′	For vector with barcode 5
>T5R1	5′‐ GACAAGGCTTCATCGTacgaccaacttctgcagttaag ‐3′	
>T6F1	5′‐ TGCCTGCATAGATACGgtaagtaagtatcaaggttacaagacag ‐3′	For vector with barcode 6
>T6R1	5′‐ CGTATCTATGCAGGCAacgaccaacttctgcagttaag ‐3′	
>T7F1	5′‐ GAAGTATCCGGTCATCgtaagtaagtatcaaggttacaagacag ‐3′	For vector with barcode 7
>T7R1	5′‐ GATGACCGGATACTTCacgaccaacttctgcagttaag ‐3′	
>T8F1	5′‐ TCCAAGGAAGGCTTCTgtaagtatcaaggttacaagacag ‐3′	For vector with barcode 8
>T8R1	5′‐ AGAAGCCTTCCTTGGAacgaccaacttctgcagttaag ‐3′	
>T9F1	5′‐ AGGTTATACGCCGCTAgtaagtatcaaggttacaagacag ‐3′	For vector with barcode 9
>T9R1	5′‐ TAGCGGCGTATAACCTacgaccaacttctgcagttaag ‐3′	

a Nucleotides in capital case are sequences corresponding to the barcode sequences. Nucleotides with small case are sequences matching the vector for primer pairing.

Second to facilitate the insertion of potential enhancers, we modified the MCS. Because potential enhancers are usually cloned by PCR using Taq DNA polymerase, which produces a 3′ A overhang, we inserted two *XcmI* restriction sites into the MCS (Fig. 1). *XcmI* digestion linearizes the vector and produces 3′ T overhangs compatible with T/A cloning. This allows fast cloning of PCR products directly into the reporter vector. In addition, unique *EcoRV* and *SpeI* sites were also introduced, allowing more choice for cloning.

### Detection of Enhancer Activity In Vivo

To assay enhancer activity after electroporation, we designed an RT‐PCR strategy that detects barcode‐specific transcripts driven by the potential enhancer. Transcripts from each reporter vector are detected by a barcode‐specific forward primer and a common reverse primer. To distinguish amplicons from RNA transcripts and plasmid DNA, the forward primers span the intron: each primer matches the barcodes plus an extra 4 nucleotides downstream of the intron‐exon junction (Fig. 1; blue and green line). The same reverse primer located within the EGFP coding region is used in combination with each forward primer. Thus, only RNA transcripts targeted by barcode‐specific and common reverse primers will be amplified to produce a 129‐bp‐long product.

**Figure 2:**
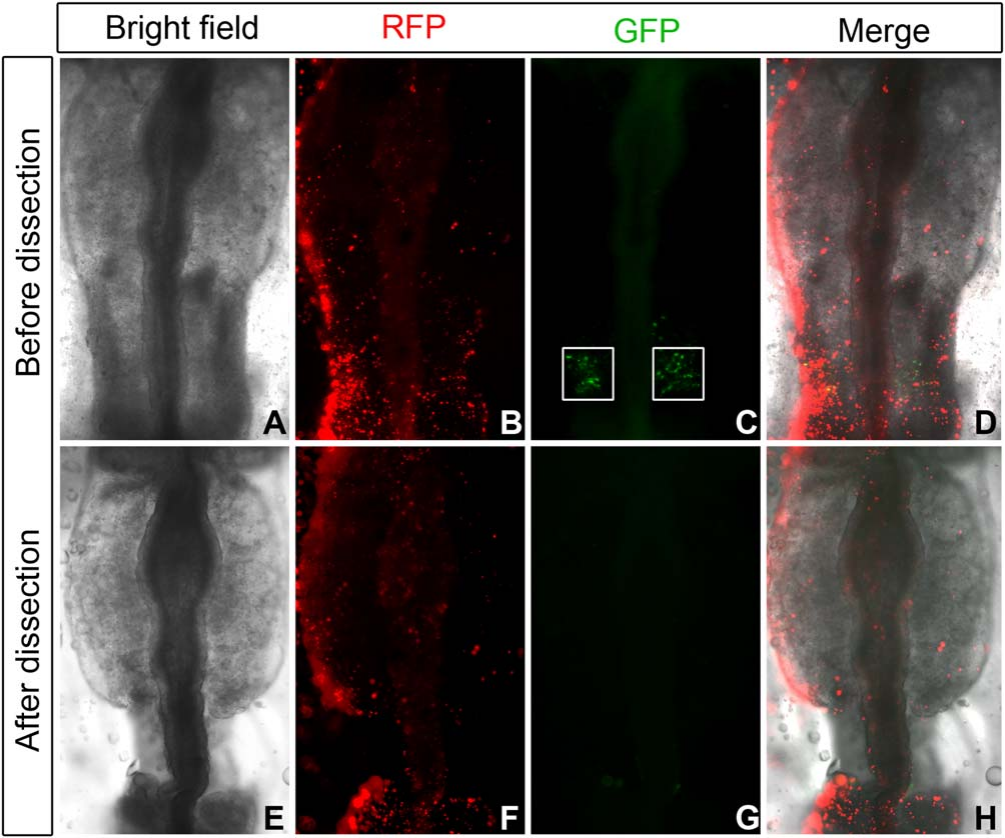
Dissection of electroporated embryos. RFP is expressed from the control plasmid using a βactin promoter and indicates the location of successful electroporation, while Sox10E‐driven EGFP is detected only in the otic placode (**A–D**). The otic placode demarcated by the white squares (C) is dissected for RT‐PCR analysis (**E–H**).

**Figure 3:**
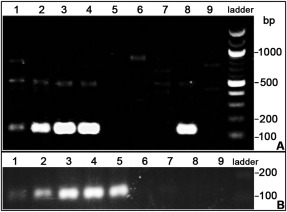
Otic enhancer detection by RT‐PCR. **A**: The activity of otic enhancers. Spalt4F14 (Barembaum and Bronner‐Fraser, 2010), mSix1‐21 1x, mSix1‐21 2x, mSix1‐21 4x (Sato et al., 2010), and Sox10E (Betancur et al., 2011) activity is detected by the ∼129‐bp amplicons, as shown in lanes 1**–**4 and 8, respectively. Negative control plasmids do not show any band (lanes 5**–**7 and 9). Unspecific bands higher than 129 bp are also present, but can clearly be distinguished from the positive signal. **B**: Non‐otic enhancers are not detected. While the otic enhancers Spalt4F14, mSix1‐21 1x, mSix1‐21 2x, mSix1‐21 4x, and Sox10E produce a specific signal of ∼129 bp (lanes 1**–**5), the non‐otic enhancers Sox2‐N2 and Sox2‐N4 (Uchikawa et al., 2003) produce no signal (lanes 8 and 9), nor do the empty vectors (lanes 6 and 7).

To test the method, we cloned various known otic enhancers: Sox10E (Betancur et al., 2011), Spalt4F14 (Barembaum and Bronner‐Fraser, 2010), and mSix1‐21 (1x, 2x, and 4x; Sato et al., 2010) into vectors containing different barcodes. These five enhancer‐containing plasmids were mixed with four barcoded empty vectors as negative control, each at a final concentration of 0.2 μg/μl. The plasmid pool was electroporated together with a control plasmid, which expresses RFP driven by the ubiquitous β‐actin promoter, into the chick head ectoderm at HH6. At HH10–12, RFP expression is observed in a large domain, while EGFP expression is confined to the otic placode driven by the known otic enhancers (Fig. 2). The otic placode was dissected (Fig. 2C, square), RNA isolated, and 2.0 ng RNA were used for RT‐PCR with unique barcode and common reverse primers. Gel electrophoresis reveals that known positive enhancers produce a clear PCR product at around 129 bp (Fig. 3A, lanes 1, 2, 3, 4, and 8), while the negative control plasmids do not (Fig. 3A, lanes 5, 6, 7, and 9). Weak unspecific bands above 500 bp are observed in both enhancer containing and negative control plasmids (Fig. 3A); however, they can clearly be distinguished from expected product of ∼129 bp.

To test whether negative enhancers produce false positive signal in the assay, we cloned two neural tube‐specific enhancers, Sox2‐N2 and Sox2‐N4 (Uchikawa et al., 2003) into vectors with different barcodes. Both plasmids were mixed with the five plasmids containing otic enhancers and two barcoded empty vectors. Otic placodes were dissected at HH10–12 and processed as described above. Consistently, known otic enhancers produced barcode‐specific transcripts as detected by RT‐PCR (Fig. 3B, lanes 1–5), while non‐otic enhancers did not produce any specific signal (Fig. 3B, lanes 8 and 9).

**Figure 4:**
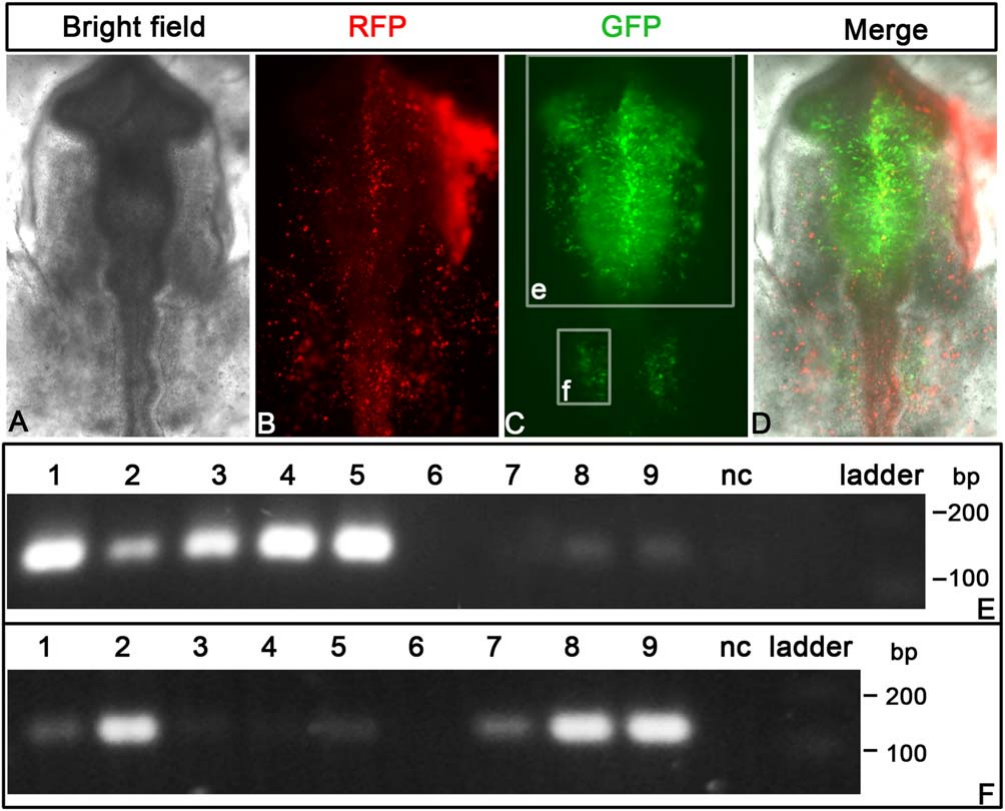
Detection of enhancers in neural tube and neural crest. **A–D**: Embryos were electroporated with a mixture of barcoded plasmids containing FoxD3‐NC1 and ‐NC2, Sox10E, Sox2‐N2, Sox2‐N4, mSix1‐21 1x/2x/4x enhancers, empty vector, and β‐actin promoter‐driven RFP at HH6/7. At HH10, enhancer activity (EGFP) is seen in the neural tube, the neural crest, and otic placode (C, D), while RFP expression is widespread. The white rectangles e and f indicate the regions dissected for assaying enhancers. **E**: Positive enhancers in the head region are detected by the 129‐bp bands (FoxD3‐NC1: lane 1, Sox10E: lane 2, Sox2‐N2: lane 3, Sox2‐N4: lane 4, FoxD3‐NC2: lane 5, Six1‐21‐2x: lane 8, Six1‐21‐4x: lane 9). Lane 6, 7, and 10 show Sox2‐N3, Six1‐21‐1x, and the empty vector, respectively. **F**: Otic enhancers are captured by the assay. Sox10E (lane 2), Six1‐21‐1x/2x/4x (lanes 7**–**9) produce the specific band around 129 bp. Lane1 (FoxD3‐NC1) and lane 5 (FoxD3‐NC2) show weak signal probably resulting from neural crest contamination. The neural tube enhancers (Sox2‐N2: lane 3, Sox2‐N4: lane 4, Sox2‐N3: lane 6) are negative in the otic placode.

To test whether this assay is useful for dispersed cells and other tissues, we cloned neural crest (FoxD3‐NC1/NC2, Sox10E; Simoes‐Costa et al., 2012; Betancur et al., 2011) and neural tube enhancers (Sox2‐N1/N2/N3; Uchikawa et al., 2003) into our barcoded vectors. These constructs were electroporated into the cranial ectoderm at HH6 together with Six1‐21 1x/2x/4x (Sato et al., 2012). As expected, the expression of EGFP was observed in both the neural tube and the neural crest at HH10 (Fig. 4A–D) and in the otic placode where Sox10E and Six1‐21 are active (Fig. 4A–D; Sato et al., 2012). We then dissected the head region rostral to the hindbrain (Fig. 4E) and the otic placodes (Fig. 4F) and assessed enhancer activity by RT‐PCR. In the head region, FoxD3_NC1/NC2, Sox2‐N2/N4, and Sox10E are positive (Fig. 4E, lanes 1–5) showing that enhancer activity can be detected even in dispersed cells. In addition, we observe a 129‐bp band for Six1‐21 2x/4x due to their weak activity in the olfactory placode (Fig. 4E, lanes 8,9; Sato et al., 2012) demonstrating that this method is sufficiently sensitive to detect activity in a small proportion of tissue. In contrast, Sox2‐N3 is not active at this stage (Fig. 4E, lane 6) in accordance with published data (Uchikawa et al., 2003). When the otic placode was assayed, Sox10E and Six1‐21 (1x/2x/4x) were detected as positive (Fig. 4F, lanes 2, 8, 9; see also Fig. 3). In addition, FoxD3‐NC1/NC2 also produced a weak signal (Fig. 4F, lanes 1 and 5) presumably due to the presence of few migrating neural crest cells surrounding the otic placode. Consistent with our previous results, Sox2‐N2/N4 (see Fig. 3B, lanes 8 and 9) and Sox2‐N3 are inactive in the otic placode (Fig. 4F, lanes 3, 4, 6; see Uchikawa et al., 2003).

In summary, the strategy described here allows the detection of enhancers that are active in small, dissected tissue samples (otic placode) and in a mixture of different tissues (neural tube, neural crest, head mesenchyme). It is sufficiently sensitive to capture enhancer activity in dispersed cells (neural crest cells) and in a small proportion of cells (olfactory placode).

### Sensitivity of Enhancer Detection In Vivo

**Figure 5:**
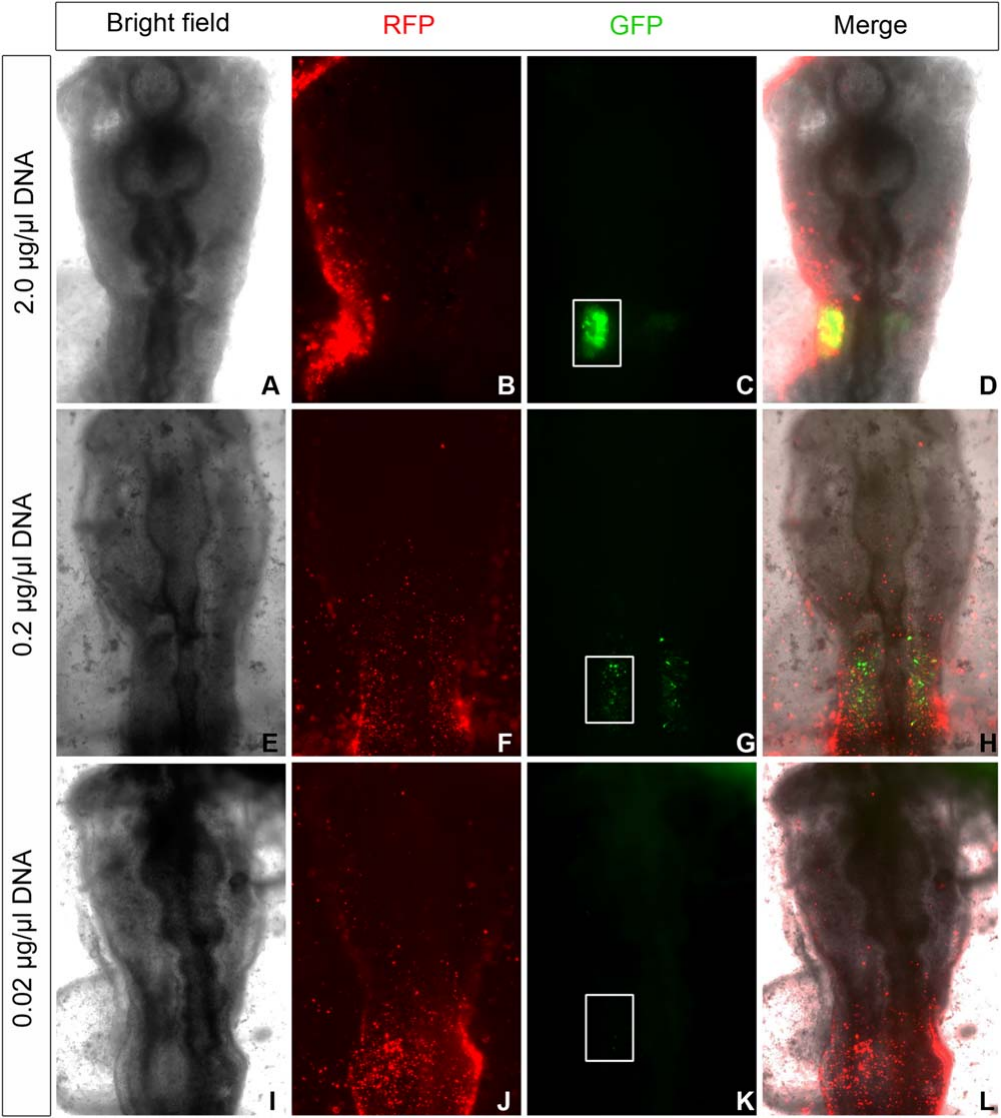
EGFP reporter detection in vivo. The Sox10E enhancer construct (Betancur et al., 2011) was electroporated into the otic region at a concentration of 2, 0.2, and 0.02 μg/μl, respectively. **A**, **E** and **I** show bright field images of electroporated embryos. RFP expression driven by the ubiquitous chick β‐actin promoter (**B, F, J**) indicates widespread electroporation, while enhancer activity is observed only in the otic placode (**C, G**). White box in C, G, and **K** indicates the otic region.

To assay many enhancer constructs in the same tissue, it is essential to detect low amounts of transcripts. To assess the sensitivity of the assay, we diluted the Sox10E plasmid to a final concentration of 2.0 μg/μl, 0.2 μg/μl, and 0.02 μg/μl, and electroporated the plasmid into the otic placode as described above. In vivo, intense otic EGFP expression is observed using the two top concentrations of Sox10E plasmid, whereas plasmids at 0.02 μg/μl yield virtually no detectable EGFP (Fig. 5).

**Figure 6:**
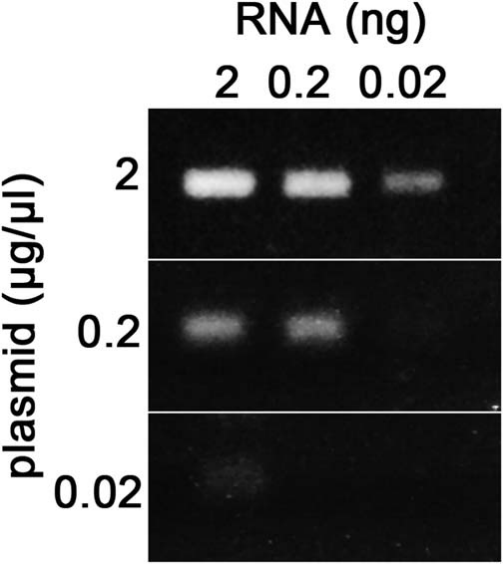
Assay sensitivity. When 2 μg/μl Sox10E reporter plasmid is used for electroporation, positive signals are detected with RNA amounts as low as 0.02 ng. When 0.2 μg/μl Sox10E reporter plasmid is used, a band is only detected with 0.2 ng RNA, while only a very faint signal is produced with 0.02 μg/μl plasmid and 2 ng RNA.

Next, we used RT‐PCR to assess the sensitivity of our method. First, we isolated total RNA from the EGFP + otic placode after electroporation of 2.0 μg/μl plasmid; 2, 0.2, and 0.02 ng RNA were used for RT‐PCR. A 129 bp band was detected with the lowest amount of RNA (Fig. 5). Using electroporation of 0.2 μg/μl plasmid required at least 0.2 ng RNA to yield a detectable signal (Fig. 6). Consistent with the faint EGFP expression after electroporation of 0.02 μg/μl plasmid DNA, only a very faint band was observed after RT‐PCR (Fig. 6). Based on these observations, we suggest that individual reporter plasmids for electroporation should at least have a concentration > 0.02 μg/μl. In order to detect some weak enhancers, higher concentration such as 0.1–0.2 μg/μl will be required.

## Discussion

Understanding the regulation of gene expression is central to understanding many biological processes including the mechanisms that control development, disease, and tissue regeneration. Non‐coding regions of the genome harbour enhancer elements, which are often conserved across species and provide key elements that regulate cell‐ and tissue‐specific gene expression. Over the last decade, new bioinformatics approaches have become powerful tools to predict regulatory regions, while genome‐wide experimental approaches have led to the discovery of vast numbers of putative enhancer elements. These findings provide a good resource to uncover gene regulatory networks that underlie both development and disease. However, verification of the in vivo activity of such enhancers still remains the bottleneck in amniote species. While large‐scale in vivo enhancer validation has been very successful in invertebrates like Drosophila and sea urchin (Nam and Davidson, 2012; Gisselbrecht et al., 2013), progress has been slower in amniote species due to laborious process to generate transgenic animals. Recently, parallel sequencing was employed to detect the activity of thousands of short 100–200‐bp DNA sequences (Patwardhan et al., 2012; Kheradpour et al., 2013). Despite this impressive capacity, this approach is mainly applied to cultured cells in vitro.

Therefore, it is desirable to develop a rapid, cost‐effective medium throughput strategy to test enhancer activity in amniotes. The chick provides a good developmental system, because it is easily accessible at many stages, lends itself to in vivo manipulation including electroporation of reporter constructs, and results can be obtained within a few days. Traditionally, fluorescent proteins like eGFP, RFP, CFP, or YFP are used as reporters, thus limiting the number of enhancers that can be tested in a single embryo to a few. Here, we have combined established electroporation approaches with a barcoding system that identifies transcripts specific to each enhancer. Specifically, to distinguish electroporated plasmid DNA from the enhancer‐driven transcript, the barcode sequence is separated from the GFP reporter sequence through one intron (see Fig. 1). This medium throughput strategy allows the detection of up to nine different enhancers in tissue collected from only one or two embryos, in this case from two otic placodes (approximately 2,000–3,000 cells yielding around 20 ng total RNA). It is reasonably sensitive given that transcripts can be detected from plasmids electroporated at a concentration of only 0.02 μg/μl, and importantly there is virtually no background for enhancers that are inactive. We expect that in the future this approach can be scaled up further to detect the activity of 15–20 reporter constructs.

Electroporation of plasmid DNA at a very high concentration can cause malformations in the embryo. In our experience, the concentration should be less than 6 μg/μl, with an optimum of 2–3 μg/μl. Even under this condition, it is possible to increase the throughput further by increasing the number of electroporated cells. For example, compared to the small size of the otic placode used here, a much larger area can be targeted in the developing neural tube. In this case, if total RNA recovered is increased to about 500 ng, using ∼10 ng RNA for each PCR reaction will allow testing about 50 barcoded enhancer plasmids per assay. Since results can generally be obtained within two days after electroporation, this strategy provides a rapid, cost‐efficient, and scalable method to test enhancer activity in the living embryo and quickly select enhancers for further investigation.

The method described here is also useful to examine enhancer activity over time as different tissues can be harvested at different developmental stages. Plasmid‐driven expression of a transgene is generally observed for about 2 days or more (Nakamura and Funahashi, 2001), allowing the examination of enhancers throughout this period. However, as cells divide plasmids are diluted. Therefore, to assay enhancer activity at late developmental stages stable integration of reporters using systems like Tol2 transposition (Sato et al., 2007) is required and our barcode strategy can be adapted using suitable vectors. In this case, transfection is carried out at early stages to target a large number of cells and embryos are cultured in ovo to test enhancers later.

Here, we use electroporation for embryo transfection, a method widely used to target epithelial tissues like the early epiblast or ectoderm before or during gastrulation (EGX‐XIV, HH2‐8; Cui et al., 2006; Voiculescu et al., 2008), mesoderm and endoderm before ingression (HH2‐5; Sweetman et al., 2008; Voiculescu et al., 2008), the neural tube (HH9 onwards; Sakamoto et al., 1998; Nakamura et al., 2000; Croteau and Kania, 2011), or somites (HH9‐18; Scaal et al., 2004). Thus, enhancer activity in cells and tissues derived from these regions can be validated by our assay. However, different tissues will require different transfection strategies including lipofection, which has been used for the hypoblast a pre‐gastrulation stages (Albazerchi et al., 2007), or sonoporation, which has successfully been used for example for limb mesenchyme (Ohta et al., 2008). The vectors described here are suitable for these transfection approaches.

In summary, we have developed a relatively quick and sensitive assay to detect the activity of predicted enhancers in an amniote model, the chick embryo. This allows efficient validation of enhancers acquired from bioinformatics predictions and genome‐wide experiments such as CHIPseq.

## Experimental Procedures

### Plasmid Modification

The 16‐bp DNA barcodes were inserted into the original pTK‐EGFP vector (Uchikawa et al., 2003) by PCR to replace the original 16 nucleotides at the 3′ end of exon1 (Fig. 1) with a 16‐bp barcode following the method as described (Li et al., 2011). PCR primers are listed in Table 1. PCR reaction was set up containing 10 ng pTK‐EGFP vector as template, 10 μl 5X Phusion HF buffer (NEB), 0.5 μl phusion DNA polymerase (NEB), 5 µM primer pair, 200 μM dNTPs, and water up to 50 μl reaction volume. The cycling conditions were 98°C for 30 sec followed by 20 cycles of 98°C for 10 sec, 60°C for 20 sec, and 72°C for 100 sec, and 72°C for 10 min. Dpnl (1 µl) was added to the PCR product and the reaction was incubated for 30 min at 37°C to digest the original vector. PCR product (3 μl) was transformed into the DH5α competent cells. To introduce the modified multiple cloning site, the original pTK‐EGFP vector was digested with *KpnI* and *XhoI*, and annealed double oligonucleotides containing sequences of the new multiple cloning sites (sense strand: 5′‐ CCATGGATATCATGGCCAACTGACTAGTGGC‐3′, antisense strand: 5′‐ TCGAGCCACTAGTCAGTTGGCCATGATATCCATGGGTAC‐3′) were ligated to the linearized vector. All constructs were verified by sequencing.

### Electroporation and Embryo Culture

Fertilized hens' eggs (Winter farm) were incubated at 38°C until they had reached stage HH 6 (Hamburger and Hamilton, 1951). Embryos were collected in Tyrode's saline on filter paper rings (Chapman et al., 2001) and electroporated using five 50‐ms pulses of 4 V, at an interval of 750 ms using an OvoDyne electroporator (IntraCel). For electroporation, barcode‐containing plasmids (final concentration of 0.2 µg/µl each) were mixed with control plasmid (pActB‐RFP; 1.0 µg/µl) and 0.1% fast green. Embryos were cultured until HH9–10 for assaying the enhancers of cranial neural tube and neural crest, and HH10–12 for assaying enhancers of otic placode. For the cranial neural tube and neural crest, the entire head region rostral to the hindbrain was collected. For the otic placode, fluorescent placodes were freed from underlying mesoderm and dissected from the ectoderm using steel needles.

### One‐Step RT‐PCR

RNA was extracted with RNAqueous micro total RNA isolation kit (AM1931, Life Technologies) following the manufacturer's instruction, and eluted in 20 μL elution buffer. One‐step RT‐PCR was performed using a Qiagen one‐step RT‐PCR kit (210212, Qiagen) in a total volume of 10 µl with primers listed in Table 2. PCR was performed with 35 cycles following the manufacturer's protocol. RT‐PCR was set up using 2 μl total RNA, 2 μl 5x RT‐PCR buffer, 0.2 µM dNTPs, 0.6 µM primer pair, 0.4 µl enzyme mix, 5U Recombinant RNasin® Ribonuclease Inhibitor; water was added up to 10 µl reaction volume. The cycling conditions were as follows: 50°C for 30 min, 95°C for 15 min, and 35 cycles of 94°C for 30 sec, 65°C for sec, 72°C for 30 sec, and 72°C for 10 min. PCR products were analysed on 1.5% agarose gels and imaged.

**Table 2:** Primers for RT‐PCR^a^

	Primers	Comments
>1F	5′‐CAGTTTTCAAGCCGGAgtgt‐ 3′	For vector with barcode 1
>2F	5′‐ TGATACACCGAGTCGTgtgt‐ 3′	For vector with barcode 2
>3F	5′‐ AGCTCTTCGCAAAGTGgtgt‐ 3′	For vector with barcode 3
>4F	5′‐ CAGCTTACTCGTAAGGgtgt‐ 3′	For vector with barcode 4
>5F	5′‐ ACGATGAAGCCTTGTCgtgt‐ 3′	For vector with barcode 5
>6F	5′‐ TGCCTGCATAGATACGgtgt‐ 3′	For vector with barcode 6
>7F	5′‐ GAAGTATCCGGTCATCgtgt‐ 3′	For vector with barcode 7
>8F	5′‐ TCCAAGGAAGGCTTCTgtgt‐ 3′	For vector with barcode 8
>9F	5′‐ AGGTTATACGCCGCTAgtgt‐ 3′	For vector with barcode 9
>Rev	5′‐ GTCCAGCTCGACCAGGATG‐ 3′	Universal reverse primer

a For forward primers, nucleotides in capital case are sequences corresponding to the barcode sequences.
